# A joint adventure of Sino-German researchers to explore the wild world of RNAs

**DOI:** 10.1093/jmcb/mjz097

**Published:** 2019-10-22

**Authors:** Albrecht Bindereif, Zefeng Wang

**Affiliations:** 1 CAS Key Laboratory of Computational Biology, Biomedical Big Data Center, CAS-MPG Partner Institute for Computational Biology, Shanghai Institute of Nutrition and Health, Chinese Academy of Sciences, Shanghai 200031, China; 2 University of Chinese Academy of Sciences, Chinese Academy of Sciences, Shanghai 200031, China; 3 CAS Center for Excellence in Molecular Cell Science, Chinese Academy of Sciences, Shanghai 200031, China; 4 Department of Biology and Chemistry, Institute of Biochemistry, Justus Liebig University of Giessen, Giessen D-35392, Germany

NA biology has developed in recent years to a new emerging field, with new surprises and insights ranging from basic mechanisms to topics of clinical interest and opening up biotechnological applications in molecular medicine and novel therapeutic options. Here, we edit this Special Issue of *Journal of Molecular Cell Biology* on RNA biology, combining a series of reviews and research articles that reflect most of the current trends and topics emerging from recent Chinese–German interactions in RNA research.

The Sino-German Symposium on RNA Biology has been held since 2016, each year concentrating around a special theme of RNA research. The symposium is funded by the Sino-German Center for Research Promotion, a joint venture between the Deutsche Forschungsgemeinschaft (DFG) and the National Natural Science Foundation of China (NSFC), which supports scientific collaborations between China and Germany, in particular workshop-type meetings with ~40 scientists from both countries, all presenting their latest work, discussing ongoing projects, and initiating new co-operations. Renowned scientists from other countries were also invited, such as Prof. Xiangdong Fu from UCSD, USA. Albrecht Bindereif and Zefeng Wang co-organized the first two meetings, which were held in the castle of Rauischholzhausen (Germany; July 2016) focusing on the role of RNA in human disease and at the Chinese Academy of Sciences (CAS) campus in Shanghai (China; November 2017) on RNA-related complex structures and novel mechanisms, respectively. The 3rd meeting co-organized by Markus Landthaler from the Max-Delbrück-Center for Molecular Medicine (MDC) in Berlin and Wei Chen from the Southern University of Science and Technology (SUSTech) in Shenzhen was held last year in Berlin (Germany; August 2018) and emphasized emerging technologies and new trends in RNA research. The 4th meeting will soon take place in Shenzhen in November 2019, continuing this short but successful history.

These Sino-German RNA meetings also rely on a core of long-standing interactions, collaborations, and personal friendships, some extending over the last two decades. For instance, Wei Chen spent many years in Berlin (first at the Max-Planck Institute for Molecular Genetics, then at the MDC), with a long history of successful German–Chinese collaborations; Jingyi Hui once worked in Albrecht Bindereif’s group in Berlin and Giessen before moving back to Shanghai; and Zefeng Wang co-organized the Otto-Warburg Summer Schools alternating between Berlin and Shanghai and recently became the Mercator Fellow of a DFG-funded Research Training Group coordinated by Albrecht Bindereif. In addition, new collaborations were initiated during these meetings, reflecting the need for an interaction forum for German and Chinese scientists in RNA research. We are very grateful to the Sino-German Center for Research Promotion (Beijing) for continuous support and funding to make this possible.

The collected 11 papers in this Special Issue were contributed by participants of the Sino-German Symposia since 2016. They cover diverse topics and different biological systems and are briefly clustered into two groups. The first group of articles are related to the biology of long non-coding RNA (lncRNA), with the following topics: RNA structural biology (Jones and Sattler, 2019), aging and disease aspects of lncRNA (Lozano-Vidal et al., 2019), the roles of lncRNA processing in gene expression (Ntini and Marsico, 2019), RNA polymerase III-derived non-coding RNAs and their functions (Täuber et al., 2019), circular RNA regulation during hypoxia (Di Liddo et al., 2019), and functions of lncRNAs in cancer (Han et al., 2019). The second group of articles are related to the general processing of RNAs, with topics ranging from m^6^A RNA modification in plants (Reichel et al., 2019), new insight on cap-independent translation (Yang and Wang, 2019), regulation of alternative splicing in breast cancers (Yang et al., 2019), general

mechanisms of auto-regulation by RNA-binding proteins (Müller-McNicoll et al., 2019), as well as novel roles of IDH1 in regulating translation initiation (Liu et al., 2019). In particular, one of the authors, Lichao Liu from Xiaohua Shen’s group, hand-drew a watercolor art for the cover of this Special Issue. This drawing was inspired by a legendary hero in Chinese mythology, Ne Zha, who has three heads and six arms to handle different weapons and tools simultaneously, just like the multitasking IDH1 protein (Liu et al., 2019). This same mentality of multitasking and the diverse types of research tools are exactly what we need in exploring the wild world of RNA, which we are excited to venture into with our friends.
